# Rift Valley Fever Virus Infection Causes Acute Encephalitis in the Ferret

**DOI:** 10.1128/mSphere.00798-20

**Published:** 2020-10-28

**Authors:** Dominique J. Barbeau, Joseph R. Albe, Sham Nambulli, Natasha L. Tilston-Lunel, Amy L. Hartman, Seema S. Lakdawala, Ed Klein, W. Paul Duprex, Anita K. McElroy

**Affiliations:** a University of Pittsburgh, School of Medicine, Department of Pediatrics, Division of Pediatric infectious Disease, Pittsburgh, Pennsylvania, USA; b University of Pittsburgh, School of Public Health, Department of Infectious Disease and Microbiology, Pittsburgh, Pennsylvania, USA; c University of Pittsburgh, Center for Vaccine Research, Pittsburgh, Pennsylvania, USA; d University of Pittsburgh, School of Medicine, Division of Laboratory Animal Research, Pittsburgh, Pennsylvania, USA; e University of Pittsburgh, School of Medicine, Department of Microbiology and Molecular Genetics, Pittsburgh, Pennsylvania, USA; University of Kentucky College of Medicine

**Keywords:** Rift Valley fever virus, encephalitis, ferret, animal model, pathogenesis

## Abstract

Rift Valley fever virus (RVFV) is a pathogen of both humans and livestock in Africa and the Middle East. Severe human disease is associated with hepatitis and/or encephalitis. Current pathogenesis studies rely on rodents and nonhuman primates, which have advantages and disadvantages. We evaluated disease progression in Mustela putorius furo (the ferret) following intradermal (i.d.) or intranasal (i.n.) infection. Infected ferrets developed hyperpyrexia, weight loss, lymphopenia, and hypoalbuminemia. Three of four ferrets inoculated intranasally with RVFV developed central nervous system (CNS) disease that manifested as seizure, ataxia, and/or hind limb weakness at 8 to 11 days postinfection (dpi). Animals with clinical CNS disease had transient viral RNAemia, high viral RNA loads in the brain, and histopathological evidence of encephalitis. The ferret model will facilitate our understanding of how RVFV accesses the CNS and has utility for the evaluation of vaccines and/or therapeutics in preventing RVFV CNS disease.

**IMPORTANCE** Animal models of viral disease are very important for understanding how viruses make people sick and for testing out drugs and vaccines to see if they can prevent disease. In this study, we identify the ferret as a model of encephalitis caused by Rift Valley fever virus (RVFV). This novel model will allow researchers to evaluate ways to prevent RVFV encephalitis.

## Abstract (precis)

Animal models of viral disease are very important for understanding how viruses make people sick and for testing out drugs and vaccines to see if they can prevent disease. In this study, we identify the ferret as a model of encephalitis caused by Rift Valley fever virus (RVFV). This novel model will allow researchers to evaluate ways to prevent RVFV encephalitis.

## INTRODUCTION

Rift Valley fever (RVF) is a disease endemic to Africa and the Arabian Peninsula, affecting livestock and humans (1–3). Livestock are infected via the bite of mosquitos carrying RVF virus (RVFV), a bunyavirus in the family *Phenuiviridae*. Transmission to humans can occur by mosquito bite or exposure to infected animal fluids (4). While the majority of human RVF cases result in a self-limiting, febrile illness, a subset of cases progress to severe clinical manifestations (5, 6). These include hepatitis, retinitis, encephalitis, and hemorrhagic fever, with case fatality rates varying widely between outbreaks (7). While severe disease is associated with uncontrolled viral replication and an exaggerated host inflammatory response (8, 9), the host determinants of clinical outcome are not yet defined (10). Our understanding of RVFV pathogenesis and disease progression is impeded by limitations of current animal models to faithfully recapitulate the range of human clinical manifestations.

Commonly used animal models for RVF include rodents and nonhuman primates (NHPs) (11, 12). Wild-type (WT) RVFV is typically lethal in all mouse strains tested to date, typically from acute, early-onset hepatitis (13, 14). BALB/c mice sometimes survive longer before succumbing to neurological disease (15). Inbred rats provide a wider range of susceptibility and disease progression, depending on strain and infection route (16, 17). Wistar-Furth and Brown Norway rats are highly susceptible and succumb to acute hepatitis regardless of the infection route (18), while August-Copenhagen-Irish rats skew toward encephalitis (19). Conversely, RVF manifestations in Lewis rats are highly dependent on route of infection: subcutaneously infected animals develop viremia but no disease signs (18), while aerosol exposure leads to uniformly lethal encephalitis (17, 20, 21). Syrian hamsters develop acute liver disease following subcutaneous exposure to RVFV and have also been used to evaluate vaccines and therapeutics against RVFV (22–24). However, for licensure of vaccines or therapeutics in the absence of human clinical trial data, the Food and Drug Administration (FDA) two-animal rule requires efficacy in two animal models, with at least one nonrodent system (25, 26). Nonhuman primate studies more closely recapitulate human RVF disease phenotypes (27–29), but high cost renders NHPs intractable for early-stage development, large-scale, or high-throughput studies. Therefore, a nonrodent, non-NHP animal model for RVFV that exhibits a spectrum of clinical manifestations would be a very useful addition.

Domestic ferrets (Mustela putorius furo) are a commercially available, outbred species that successfully models aspects of clinical disease for many viruses (30), including other hemorrhagic fever viruses (31–33). Ferrets are less expensive than NHPs and do not require the same large-animal facilities as NHPs but have been shown to similarly reproduce aspects of viral disease progression (31). Additionally, since ferrets are excellent models of influenza, many new virologic and immunologic reagents are actively being developed to make this a more useful resource for studying infectious diseases (34). RVFV was first shown to cause pathology in ferrets in 1935 (35). However, ferrets have not since been investigated to study RVFV pathogenesis. Here, we establish RVFV models of self-limited acute febrile disease and neurological disease in ferrets.

## RESULTS

### RVFV infection of ferrets results in a self-limited febrile illness or encephalitis.

**FIG 1:**
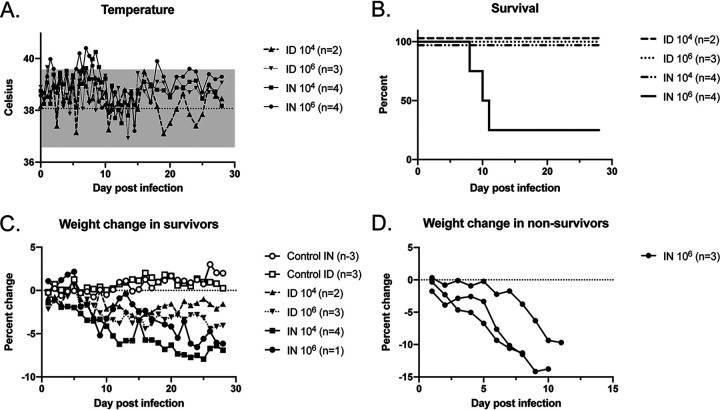
Clinical findings in RVFV-infected ferrets. Temperature measurements (A), survival (B), and weight changes (C and D) in ferrets inoculated with two different doses of rWT RVFV via the i.n. or i.d. route are shown. Numbers of animals in each group are noted. For panel A, the dotted line denotes the average of the values obtained in control animals over the course of the experiment, and the gray shading denotes 2 standard deviations from the averages.

Ferrets were infected with either 1 × 10^4^ (low dose) or 1 × 10^6^ (high dose) 50% tissue culture infective doses (TCID_50_) of recombinant WT (rWT) ZH501 RVFV via either the intradermal (i.d.) or intranasal (i.n.) route. The ferrets i.n. and i.d. infected at the high dose developed transient temperature elevation above the range of the controls in the first week of infection. Ferrets infected i.n. at both inoculation doses had temperature elevation above the range of the controls at 6 to 8 days postinfection (dpi) (Fig. 1A). The clinical scoring system recorded posture, activity, skin/eye, neurologic, pulmonary, or gastrointestinal tract findings (see Fig. S1 in the supplemental material). Control and low-dose i.d. infected animals had clinical scores of zero throughout the course. High-dose i.d. infected animals had 1 to 2 episodes of diarrhea, and one of three animals displayed intermittent head tilt from days 10 to 21 without other clinical signs. Low-dose i.n. infected animals exhibited intermittent decreased activity at 6 to 13 dpi; two of the four animals had diarrhea and squinting, with one also displaying mild respiratory distress and sneezing during this time. The high-dose i.n. infected group exhibited decreased activity, squinting, intermittent sneezing, and mild respiratory distress from 6 to 11 dpi. One of the four animals in this group had resolution of symptoms while the other three were euthanized on days 8, 10, and 11 for central nervous system (CNS) signs that included ataxia, seizures/tremors, and hind limb weakness (Fig. 1B). Throughout the experiment, most infected ferrets had weight loss compared to weight of the control animals; weight loss was greatest in animals that required euthanasia for CNS disease (Fig. 1C and D; Fig. S2). Notably, the same animal that had the head tilt in the group infected i.d. at 10^6^ TCID_50_ also had >10% weight loss (Fig. S2).

10.1128/mSphere.00798-20.1 FIG S1 Clinical scoring system. Download FIG S1, TIF file, 0.01 MB. Copyright © 2020 Barbeau et al. 2020 Barbeau et al. This content is distributed under the terms of the Creative Commons Attribution 4.0 International license.

10.1128/mSphere.00798-20.2 FIG S2 Weight and temperature change for each animal in the study. Weight change over time in control animals (A), animals that were inoculated intranasally (i.n.) (B), and animals that were inoculated intradermally (i.d.) (D) is shown. Temperature change over time in i.n. (C) and i.d. (E) inoculated animals is shown. Each animal is indicated by a unique color and shape. Gray shading indicates the range of temperatures observed in control animals. Open symbols in panel C represent animals that were euthanized for clinical disease. Individual weights are not shown for these animals since these data are depicted in Fig. 1D of the manuscript. Download FIG S2, TIF file, 0.9 MB. Copyright © 2020 Barbeau et al. 2020 Barbeau et al. This content is distributed under the terms of the Creative Commons Attribution 4.0 International license.

### RVFV-infected ferrets have mild changes in their leukocyte percentages and clinical chemistries.

**FIG 2:**
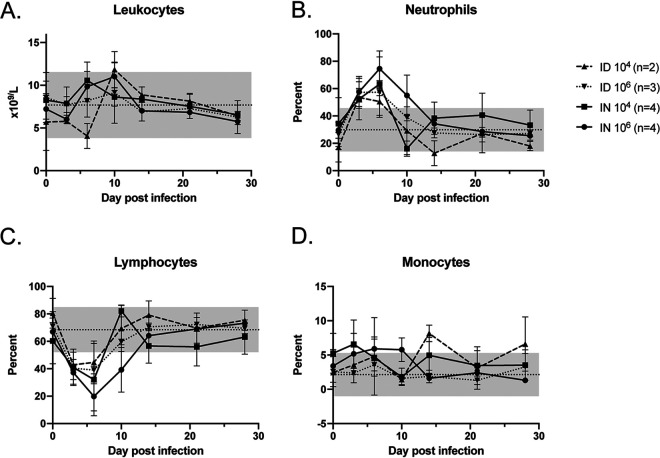
Leukocyte changes in RVFV-infected ferrets. Total leukocytes (A) and the percentages of neutrophils (B), lymphocytes (C), and monocytes (D) are shown. Dotted lines denote the averages of the values obtained in control animals over the course of the experiment, and the gray shading in each graph denotes 2 standard deviations from the averages. Error bars indicate the standard deviations of the means.

**FIG 3:**
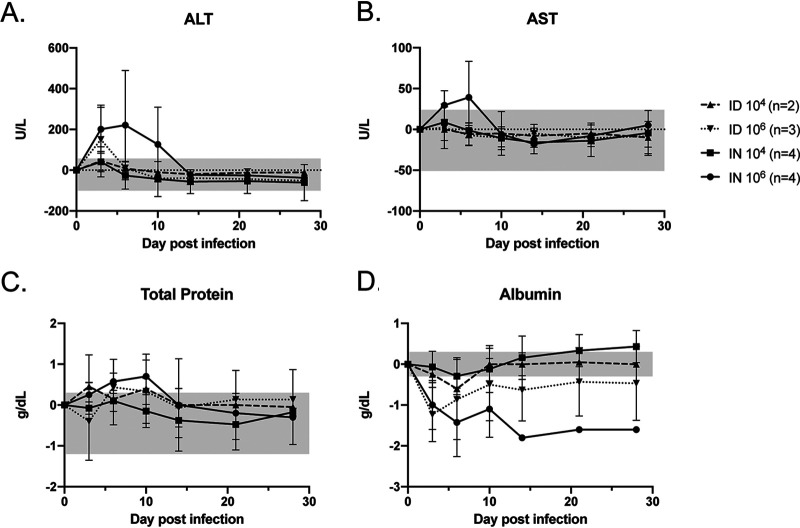
Blood chemistry changes in RVFV-infected ferrets. Measurements of liver function (ALT and AST), total protein, and albumin, as indicated, are shown as a function of change from baseline. The gray shading in each graph denotes the range of changes observed from baseline in the control animals. Error bars indicate the standard deviations of the means.

Serial complete blood counts (CBCs) revealed a relative lymphocytopenia and neutrophilia during the first week of infection that resolved in all animals (Fig. 2A to D). A transient mild elevation in levels of aspartate aminotransferase (AST) and alanine aminotransferase (ALT) were noted in the high-dose i.n. infected group, and a transient mild elevation in ALT was noted in the high-dose i.d. infected group (Fig. 3A and B). Animals infected at the high dose had an early increase in total protein (Fig. 3C) but low levels of albumin, which is the main protein component in blood and an acute-phase reactant. Hypoalbuminemia was sustained throughout the experiment in the high-dose i.n. infected group (Fig. 3D).

### RVFV-infected ferrets that required euthanasia for clinical encephalitis had high viral RNA loads in the brain.

**FIG 4:**
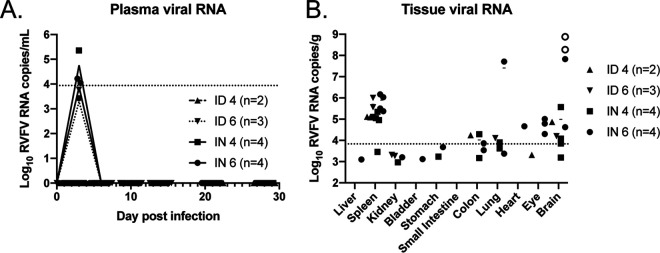
Viral RNA loads in RVFV-infected ferrets. Viral RNA levels were measured in plasma over time (*x* axis, day postinfection) (A) and in various tissues at the time of euthanasia (B). The dotted line represents the limit of detection of the assays. Open symbols indicate isolation of virus.

Transient low levels of viral RNA were noted in the blood of three high-dose i.n. infected animals at 3 dpi and in one each of the low-dose i.n. infected and high-dose i.d. infected animals (Fig. 4A). At the time of euthanasia, viral RNA was detected in the spleens of most animals but was elevated to 7 to 8 log_10_ copies per gram in the brains of the three animals that were euthanized for clinical neurologic disease (Fig. 4B). The three animals that required euthanasia also had detectable transient viral RNA in the blood at 3 dpi. One of the animals that was euthanized for neurologic disease had 7 log copies of viral RNA per gram in the lungs. No other sampled tissues exhibited notable levels of viral RNA at the time of necropsy. Isolation of infectious virus was attempted from all brain samples, from the single lung sample that had a high viral RNA load, and from the spleen samples with the three highest RNA loads. Infectious virus was isolated only from the brains of the encephalitic animals that were euthanized at 8 and 10 dpi (Fig. 4B, open circles). No virus was recovered from any sample with a viral RNA copy number of less than 8 logs. This was not surprising, given the sensitivity of the quantitative reverse transcription-PCR (qRT-PCR) assay, and similar findings have been reported by other investigators (36).

### All RVFV-infected ferrets developed virus-specific adaptive immune responses.

**FIG 5:**
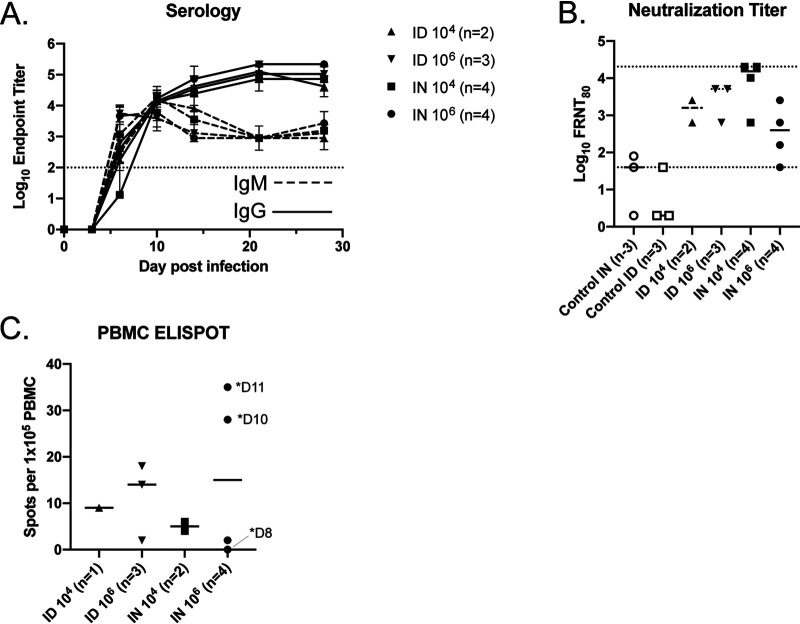
Immunologic response in RVFV-infected ferrets. Average anti-RVFV IgM (dashed lines) and IgG (solid lines) enzyme-linked immunosorbent assay endpoint titers as measured over time (A) and neutralizing antibody titers at the time of euthanasia (B) are shown. The dotted lines represent the limits of detection of the assays. Error bars indicate the standard deviations of the means. (C) Virus-specific cellular immunity as measured by ELISPOT assay. Days (D) of euthanasia are indicated for the three animals that required euthanasia.

RVFV-specific antibodies were first noted at 6 dpi (Fig. 5A). IgM titers were initially higher than those of IgG; but by 10 dpi, they were equivocal, and thereafter IgG titers continued to rise while IgM titers declined and then persisted. There was no difference in the magnitude of the virus-specific titers between the different experimental groups. All surviving RVFV-infected animals had 80% focus reduction neutralization test (FRNT_80_) values of 640 or greater while the three animals that developed encephalitis had RVFV FRNT_80_ values of 40 to 640 on the day of euthanasia, which occurred between 8 and 11 dpi (Fig. 5B).

Peripheral blood mononuclear cells (PBMCs) obtained at euthanasia were analyzed for virus-specific cellular immunity by gamma interferon (IFN-γ) enzyme-linked immunosorbent spot (ELISPOT) assay. Virus-specific cells were detected in all animals from which viable PBMCs were obtained except in the animal euthanized at 8 dpi for CNS disease (Fig. 5C). This animal had detectable spots in the peptide-exposed wells but also had detectable spots in the negative-control well, indicating nonspecific cellular activation at the time of euthanasia. The highest frequency of RVFV-specific cellular activity was noted in the two animals that were euthanized at 10 and 11 dpi.

### All i.n. RVFV-infected ferrets had histopathologic evidence of encephalitis.

**FIG 6:**
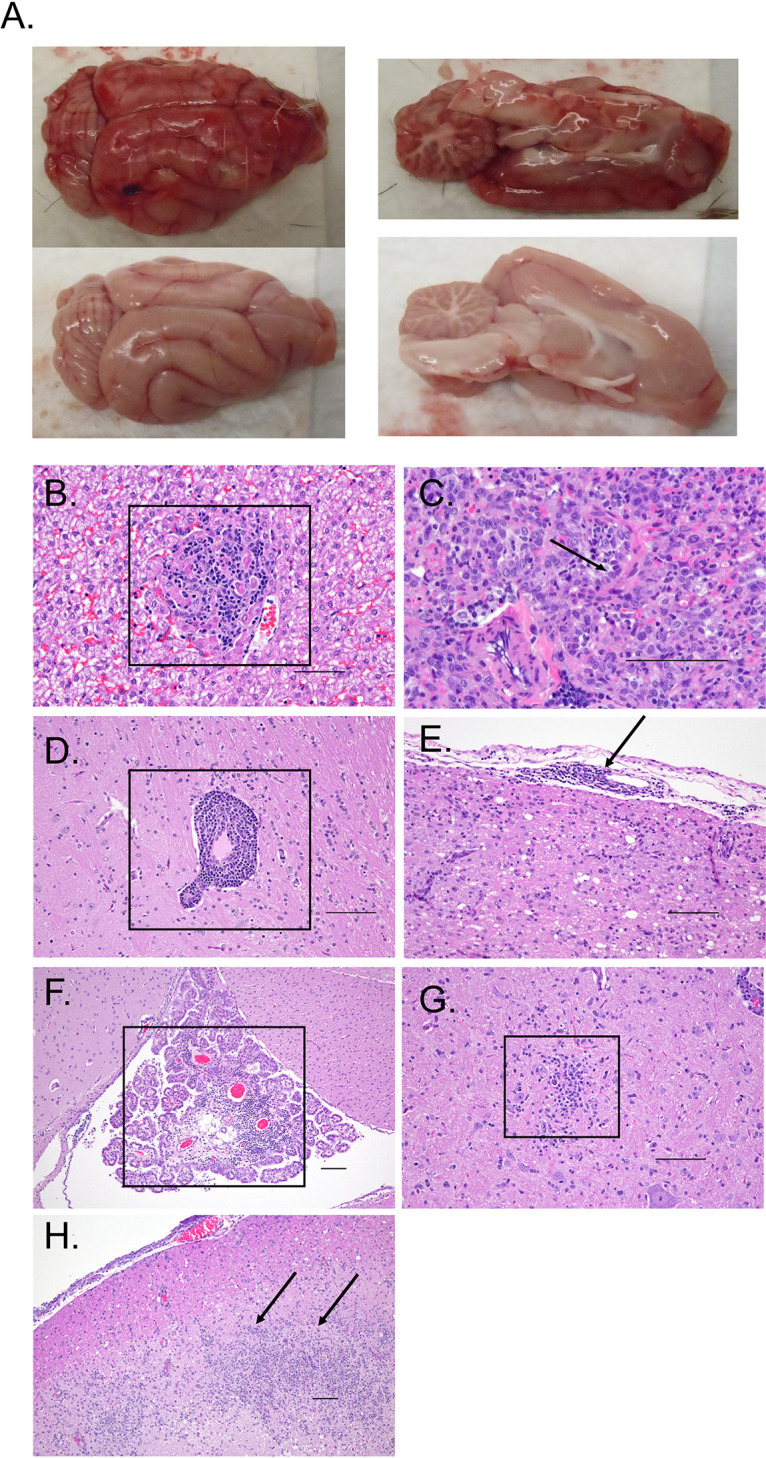
Pathologic findings in RVFV-infected ferrets. (A) Photograph of a grossly abnormal brain obtained during the necropsy of a high-dose i.n. infected ferret. The brain was characterized by diffuse hyperemia (top). An uninfected control brain is shown for comparison (bottom). (B) Foci (boxed) of hepatic inflammation and necrosis were noted in the liver of an animal infected with RVFV i.n. at 10^4^ TCID_50_ (magnification, ×20). (C) Viral RNA detection in the lungs of one animal infected with RVFV i.n. at 10^6^ TCID_50_ corresponded with diffuse pneumonitis and type II pneumocyte proliferation (arrow) in the setting of lung collapse (magnification, ×40). Perivascular infiltrates (boxed, ×20 magnification) (D), meningitis (arrow, ×20 magnification) (E), choroiditis (boxed, ×10 magnification) (F), glial nodules (boxed, ×20 magnification) (G), and laminar necrosis (arrows, ×10 magnification) (H) were noted in the brains of animals with encephalitis. Representative CNS photos are shown from animals infected with RVFV i.n. at 10^6^ TCID_50_. Scale bar, 100 μm.

An extensive necropsy was performed at the time of euthanasia. Mild splenic enlargement was noted in most of the infected animals. There were no obvious gross abnormalities of the pulmonary, urinary, or gastrointestinal tracts of any animal, with the exception of incidental renal cysts in 2 of 19 animals. In the animal that required euthanasia due to CNS disease at 8 dpi, the surface of the brain was grossly discolored, consistent with hyperemia and inflammation (Fig. 6A), and the sagittal cut revealed that the deeper neuropil was also affected (similar specimens are shown from an uninfected animal for comparison). On histopathologic examination, no significant lesions were noted in the stomach, small intestine, colon, or eyes of any animal. Several of the animals (infected and controls) were noted to have incidental findings of mild distal tubular mineralization in the kidney as well as mild patchy urothelial cell anisocytosis and syncytialization that were unlikely to be associated with RVFV infection. Infected and control animals had various degrees of mild to moderate lymphocytic periportal inflammatory infiltrates in the liver, which is not uncommon among ferrets (37). Four of the 13 infected animals had foci of mixed inflammation accompanied by residual hepatocyte necrosis (Fig. 6B). Notably, pathological findings in the liver did not correlate with dose, route, or ALT/AST elevation. The lungs from control and infected animals exhibited minimal acute patchy bronchitis of unclear significance. However, the one animal that had high levels of viral RNA in its lungs also had mild to moderate acute patchy pneumonitis with type II pneumocyte proliferation (Fig. 6C). The brains of all i.n. infected animals as well as the brain of one of the animals infected i.d. at 10^6^ TCID_50_ exhibited perivascular cuffing (Fig. 6D), multifocal patchy meningitis (Fig. 6E), and encephalitis. Additional findings present in the brains of animals euthanized for clinical CNS disease included choroiditis (Fig. 6F), glial nodules (Fig. 6G), and laminar necrotizing encephalitis (Fig. 6H). The same animal infected i.d. at 10^6^ TCID_50_ that had histopathologic evidence of encephalitis was also noted to have mild patchy myocarditis (data not shown). No other animals had any pathologic lesions in the heart.

## DISCUSSION

While several different animal models exist for studies of RVF disease, each of these models has some limitations. Most species of mice are exquisitely sensitive to RVFV infection and succumb rapidly to hepatitis. Nonhuman primates have shown promise and are excellent for later preclinical development studies but can be difficult to obtain and require significant resources. Rats have somewhat more diverse phenotypes but, as rodents, also do not meet the required FDA two-animal rule for the evaluation of therapeutics or vaccines for licensure. The two-animal rule would be a likely path of licensure of any RVFV vaccine or therapeutic given the difficulty of performing a clinical trial for a disease that exhibits sporadic emergence in wide geographic areas.

The ferret model fills this gap in that it is a nonrodent animal species that is readily available and less resource-intensive than NHPs. Additionally, with wide use of ferrets for influenza studies, there is a wealth of knowledge already in place, and new immunologic reagents are being actively developed (34). The ferret model recapitulates at least two important manifestations of RVFV disease that are seen in humans: mild self-limited febrile illness and later-onset severe encephalitis. Importantly, the RVFV CNS disease that was observed in ferrets occurred following exposures that mimic natural human routes of exposure, suggesting that the RVFV ferret model could be useful for studying how the virus gains access to the CNS following infection by mosquito bite versus that through mucosal exposure.

The host determinants of RVF disease manifestations in humans are poorly understood. There are data to suggest that host innate immune responses modulate clinical disease manifestations; polymorphisms in Toll-like receptor 3 (TLR3), TLR7, TLR8, MyD88, TRIF, mitochondrial antiviral signaling protein (MAVS), and RIG-I genes were associated with severe hemorrhagic or encephalitic disease (8). Additionally, adaptive immunity could also modulate disease in humans since HIV-1-positive individuals are more likely to suffer from encephalitis (6, 38). These phenomena are partially represented in murine models; mice with various forms of altered innate and/or adaptive immunity develop late-onset encephalitis following infection with a highly attenuated version of RVFV that has a deletion of the nonstructural small (NSs) protein, the major virulence factor (39, 40). Additionally, BALB/c mice sometimes develop late-onset encephalitis (13) although the mechanisms that modulate this phenotype are unknown.

It is also possible that the route of exposure plays an important role in modulation of disease manifestations. Humans become exposed either by an infected mosquito bite or by mucosal exposure to the blood or bodily fluids of infected animals (41). Given the proximity of a typical mucosal exposure (i.e., eyes, mouth, or nose) to the CNS, it is possible that direct neuroinvasion occurs. This is certainly the case in the rodent model since footpad and intranasal exposures of mice with an attenuated recombinant form of RVFV have divergent outcomes (42). In this model, i.n. exposure leads to CNS invasion and encephalitis while the footpad exposure leads to robust, protective immunity. In rats and NHPs, aerosol exposure is usually associated with CNS disease while a peripheral exposure is often mild and self-limiting (17, 27, 43).

The ferret pathogenesis studies presented here recapitulate both routes of exposure to RVFV, mimicking a mosquito bite-delivered inoculation or a mucosal exposure that might occur from infected livestock. Both routes of exposure resulted in transient low-level viremia accompanied by mild and transient ALT elevation and hypoalbuminemia, indicating systemic spread and inflammation. This was most prominent in the high-dose i.n. infected group. While direct spread from i.n. inoculation across the cribriform plate cannot be ruled out, the detection of viremia also suggests that systemic spread could be another route of CNS invasion by RVFV in ferrets. A role for systemic spread is further supported by the histopathologic evidence of CNS involvement in one animal that received an i.d. inoculation. This ferret model utilizes rWT RVFV of the ZH501 strain, which has been used in many other studies of RVFV (12, 17, 27, 36, 44–48) in rodent and NHP models. Importantly, in this study, virus was delivered by routes that mimic natural infection while some encephalitis models of RVFV have required attenuated virus or nonnatural delivery routes. Finally, the outbred nature of ferrets provides host genetic diversity such as that which is present in human populations, making this model useful in understanding the impact of genetic diversity on RVFV disease manifestations.

Unlike most inbred rodent models (14), ferrets did not develop severe hepatitis following RVFV infection. Intradermal and low-dose i.n. inoculation in ferrets recapitulated a typical mild human disease course as well as what has been observed in most NHP models, the notable exception being marmosets, which are more susceptible than most NHPs following multiple routes of exposure (27, 29, 43). ALT alterations noted in ferrets were milder than those reported in NHPs, and leukopenia/leukocytosis has been variably reported in NHPs. Similar to results in both rodent and NHP models, all surviving ferrets developed robust virus-specific humoral responses.

In 1935, experimental exposure of ferrets to RVFV was reported (35). In that study the exposure route was intranasal or subcutaneous, and the animals developed respiratory disease. No mention of CNS disease was reported; but as the investigators were initially expecting to find influenza, the lungs were the major focus, and no virologic or pathological data were reported from the CNS. Additionally, there were significant limitations in the available technology at the time that influenced the reported results, given that virus was assayed in mice and that mammalian cell culture had not yet been developed. The authors identified the virus as RVFV based upon histologic findings in the livers of infected mice and the observation that RVFV immune serum from a survivor was able to protect mice from challenge. Interestingly, we found high levels of viral RNA as well as histopathologic evidence of pneumonitis in the lungs of one animal at the time of euthanasia even though respiratory symptoms were mild and self-limited. This animal was euthanized for clinical CNS disease at 8 dpi. The lungs were not systematically sampled for this study; therefore, the lack of viral RNA in the lungs of the other animals could have been secondary to a sampling or timing bias.

In summary, we evaluated the pathogenesis of RVFV infection in ferrets and observed both self-limited febrile illness and acute encephalitis. These studies provide a new model for RVFV CNS disease using the outbred domestic ferret. This model is very attractive because it uses modes of exposure that mimic natural routes of infection resulting in disease that is clinically consistent with what has been reported for human RVFV disease. Ferrets will be especially useful for the evaluation of therapeutics or vaccines that aim to treat or prevent encephalitis caused by RVFV. An i.d. or intramuscular vaccination followed by a high-dose i.n. WT RVFV challenge would be a reasonable starting point. Indeed, thanks to the efforts of the Coalition for Epidemic Preparedness Innovations, there are efforts under way to develop RVFV vaccines for human use. It will be important to ensure that these vaccines can prevent RVFV encephalitis, and the ferret provides a tractable model in which to perform these studies.

## MATERIALS AND METHODS

### Animal approvals and procedures.

Institutional Animal Care and Use Committee approval was granted from the University of Pittsburgh. All work with live virus was performed in the Center for Vaccine Research Regional Biocontainment Laboratory, which is approved for work with select agents. Ferrets (Triple F Farms) were all male due to size requirements to permit repeated phlebotomy. Animal ages ranged from 6 to 9 months at the time of infection, with weights from 1,123 to 1,837 g. Animals were implanted with an IPTT-300 temperature and i.d. transponder (BMDS) and were housed in an ABSL-3 laboratory in HEPA-filtered biocontainment caging. Animals were monitored twice daily for the first 2 weeks and daily thereafter. Daily weights, temperatures, and clinical observations were recorded. Animals were anesthetized using isoflurane for cranial vena cava phlebotomy at 0, 3, 6, 10, 14, 21, and 28 days postinfection (dpi). At each phlebotomy, blood was collected for complete blood counts (CBCs) and clinical chemistries (CHEM). CBC and CHEM data were analyzed using a VETSCAN HM5 hematology analyzer (Abaxis) and a VETSCAN VS2 chemistry analyzer (Abaxis) using the Preventative Care Profile Plus disc. At the end of the experiment or when indicated by clinical signs, animals were euthanized by intravenous injection of 2 milliequivalents (mEq)/kg of potassium chloride following terminal phlebotomy under anesthesia with inhaled isoflurane. During necropsy, various tissues were collected for virologic assays in phosphate-buffered saline (PBS) supplemented with antibiotics and antimycotic (Invitrogen). Samples of tissues were also fixed in 10% formalin for pathological analysis. Fixed tissues were processed and paraffin embedded. Sections (4 μm) were cut by microtomy and mounted on glass sides, and tissues were stained with hematoxylin and eosin (H&E).

### Virus generation, propagation, and infection.

rWT ZH501 was generated using an established reverse-genetics system (49) and propagated in Vero E6 cells (ATCC) for two passages. Virus was fully sequenced using next-generation sequencing prior to use *in vivo*. Titers of viral stocks were determined using the TCID_50_ method (50). On the day of infection 1 × 10^4^ or 1 × 10^6^ TCID_50_ was inoculated either intradermally on a shaved area between the scapulae (100 μl) or intranasally (500 μl) distributed in both nares under isoflurane anesthesia.

### qRT-PCR assays and virus isolation.

Tissues were homogenized using a D2400 homogenizer (Benchmark Scientific), and RNA was extracted from tissue homogenates or plasma samples using TRIzol reagent (Ambion) and a Direct-zol RNA purification protocol (Zymo Research). Viral RNA was detected using qRT-PCR targeting the RVFV large (L) segment as previously described (51). Data were normalized by tissue weight and are reported as copies of RNA based upon a standard curve of known-quantity RVFV L segment RNA that was generated as previously described (14). Virus isolations were performed by inoculation of tissue homogenates onto Vero E6 cells. Graphs were generated using GraphPad Prism, version 8.

### Enzyme-linked immunosorbent and neutralization assays.

RVFV-specific antibody titers were assessed using a previously described protocol (14) with the following modifications. For detection of virus-specific IgG, anti-ferret IgG-horseradish peroxidase (HRP) (Novus) was used as a secondary antibody at 1:10,000. For detection of virus-specific IgM, plates were incubated for 1 h at 37°C with a goat anti-ferret IgM (Rockland) diluted 1:5,000 in blocking buffer, followed by three PBS-Tween 20 (PBST) washes and then incubated for 1 h at 37°C with rabbit anti-goat (HRP) secondary antibody (Invitrogen) diluted 1:5,000 in blocking buffer. Data were analyzed using Excel (Microsoft Corp.). Raw optical density (OD) values from the negative-control Vero E6 plate were subtracted from those of the RVFV lysate plate. The endpoint titer was defined as the dilution of the plasma that gave a value at least 3 standard deviations above the average value obtained from negative-control plasma from an uninfected ferret.

Serum samples obtained from terminal phlebotomy were used in a focus reduction neutralization test (FRNT) as previously described (52). Foci were counted in control wells and compared to foci numbers in experimental wells. The dilution of serum at which 80% of foci are neutralized is reported as the FRNT_80_. Graphs were generated using GraphPad Prism, version 8.

### ELISPOT assay.

At terminal phlebotomy, blood was collected into cell preparation tubes (BD Biosciences), and peripheral blood mononuclear cells (PBMCs) were prepared and stored in liquid nitrogen until use. Cryopreserved PBMCs were thawed and washed in RPMI medium supplemented with 10% (vol/vol) fetal bovine serum (FBS). PBMCs were incubated with no stimulation (dimethyl sulfoxide [DMSO] control), with 1 μg/ml of staphylococcal enterotoxin B (positive control), or in duplicate with a pool of peptides that represented the entirety of the RVFV nucleocapsid (N) protein. There were a total of 59 peptides, each 15 amino acids (aa) in length with overlaps of 11 aa, suspended in DMSO. Final peptide concentration was 1 μg of each peptide/ml. Serial dilutions of PBMCs mixed with the various stimulation conditions were placed in a precoated ferret interferon gamma (IFN-γ) ELISPOT plate (Mabtech). PBMCs from uninfected ferrets were used as controls in all assays. Following overnight incubation, plates were processed according to the manufacturer’s instructions. Spots were counted using an ImmunoSpot Analyzer (CTL), and data were exported to Excel. The RVFV-specific spot count was determined by subtracting signal of the no-stimulation wells from signals of the RVFV N protein peptide pool wells, and the number of spots was recorded per 1 × 10^5^ cells. Graphs were generated using GraphPad Prism, version 8.
