# Supplementary figures and images for: Rift Valley Fever Virus Infection Causes Acute Encephalitis in the Ferret
Source: mSphere. 2020 Oct 28;5(5):e00798-20. doi: 10.1128/mSphere.00798-20 (PMC7593599; doi:10.1128/mSphere.00798-20)

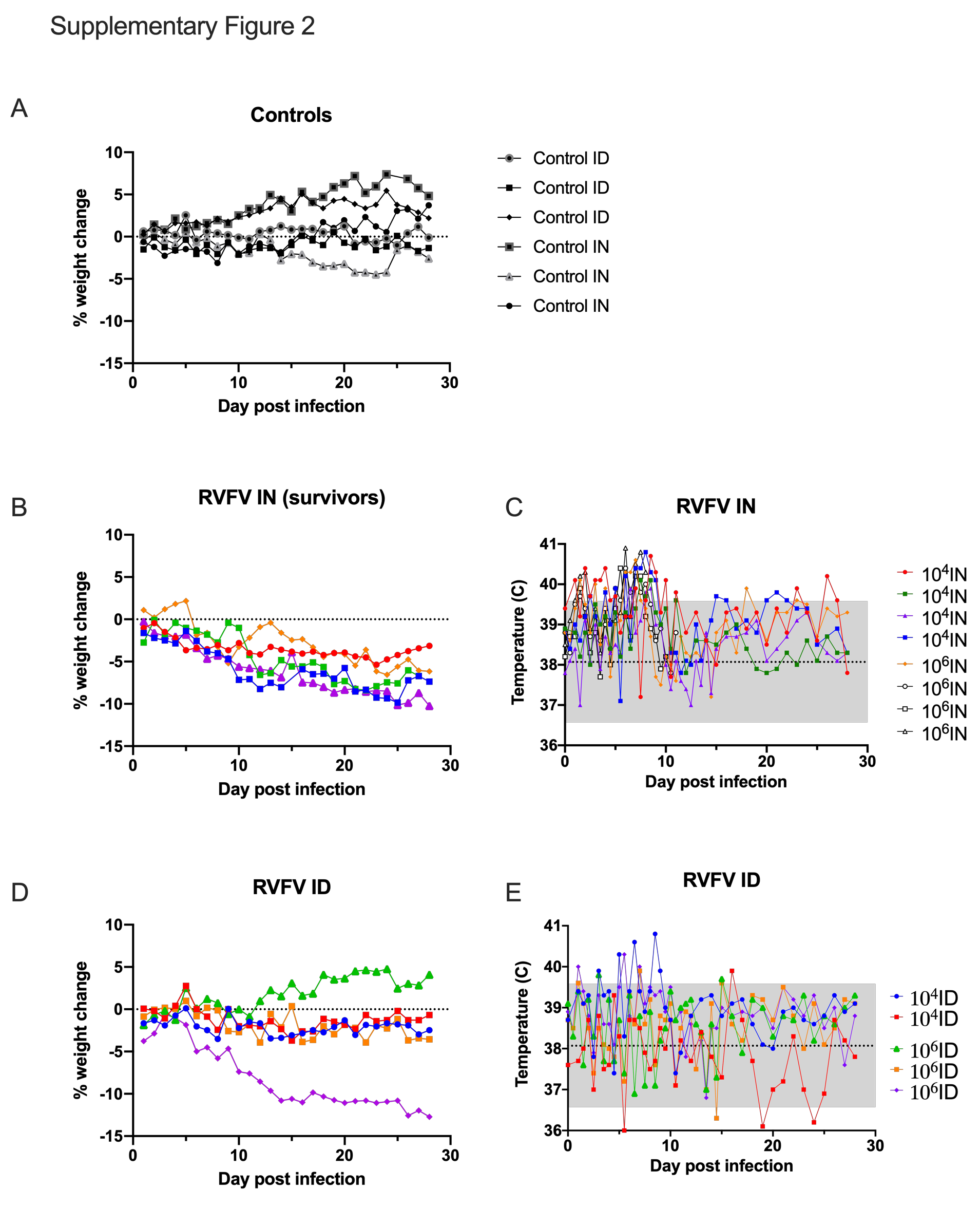

Supplement: FIG S2 [file mSphere.00798-20-sf002.tif]
